# The influence of computer-based cognitive flexibility training on subjective cognitive well-being after stroke: A multi-center randomized controlled trial

**DOI:** 10.1371/journal.pone.0187582

**Published:** 2017-11-16

**Authors:** Renate M. van de Ven, Jaap M. J. Murre, Jessika I. V. Buitenweg, Dick J. Veltman, Justine A. Aaronson, Tanja C. W. Nijboer, Suzanne J. C. Kruiper-Doesborgh, Coen A. M. van Bennekom, K. Richard Ridderinkhof, Ben Schmand

**Affiliations:** 1 Department of Psychology, University of Amsterdam, Amsterdam, The Netherlands; 2 Department of Psychiatry, VU University Medical Center, Amsterdam, The Netherlands; 3 Department of Research and Development, Heliomare Rehabilitation Center, Wijk aan Zee, The Netherlands; 4 Center of Excellence in Rehabilitation Medicine, Brain Center Rudolf Magnus, University Medical Center Utrecht, and De Hoogstraat Rehabilitation, Utrecht, The Netherlands; 5 Department of Experimental Psychology, Utrecht University, Utrecht, the Netherlands; 6 Amsterdam Rehabilitation Research Center, Reade, Amsterdam, The Netherlands; 7 Amsterdam Brain & Cognition (ABC), University of Amsterdam, Amsterdam, The Netherlands; 8 Department of Medical Psychology, Academic Medical Center, University of Amsterdam, Amsterdam, The Netherlands; University of Glasgow, UNITED KINGDOM

## Abstract

**Background:**

Stroke can result in cognitive complaints that can have a large impact on quality of life long after its occurrence. A number of computer-based training programs have been developed with the aim to improve cognitive functioning. Most studies investigating their efficacy used only objective outcome measures, whereas a reduction of subjective cognitive complaints may be equally important for improving quality of life. A few studies used subjective outcome measures but were inconclusive, partly due to methodological shortcomings such as lack of proper active and passive control groups.

**Objective:**

The aim of the current study was to investigate whether computer-based cognitive flexibility training can improve subjective cognitive functioning and quality of life after stroke.

**Methods:**

We performed a randomized controlled double blind trial (RCT). Adults (30–80 years old) who had a stroke 3 months to 5 years ago, were randomly assigned to either an intervention group (n = 38), an active control group (i.e., mock training; n = 35), or a waiting list control group (n = 24). The intervention and mock training consisted of 58 half-hour sessions within 12 weeks. The primary subjective outcome measures were cognitive functioning (Cognitive Failure Questionnaire), executive functioning (Dysexecutive Functioning Questionnaire), quality of life (Short Form Health Survey), instrumental activities of daily living (IADL; Lawton & Brody IADL scale), and participation in society (Utrecht Scale for Evaluation of Rehabilitation-Participation). Secondary subjective outcome measures were recovery after stroke, depressive symptoms (Hospital Anxiety Depression Scale—depression subscale), fatigue (Checklist Individual Strength—Fatigue subscale), and subjective cognitive improvement (exit list). Finally, a proxy of the participant rated the training effects in subjective cognitive functioning, subjective executive functioning, and IADL.

**Results and conclusions:**

All groups improved on the two measures of subjective cognitive functioning and subjective executive functioning, but not on the other measures. These cognitive and executive improvements remained stable 4 weeks after training completion. However, the intervention group did not improve more than the two control groups. This suggests that improvement was due to training-unspecific effects. The proxies did not report any improvements. We, therefore, conclude that the computer-based cognitive flexibility training did not improve subjective cognitive functioning or quality of life after stroke.

## Introduction

Up to 92% of stroke survivors report cognitive complaints in, for example, executive functioning, attention, memory, and processing speed [1]. These subjective complaints can be long lasting [2] and have been associated with lower return to work rates [3], higher mortality risk [4], worse cognitive functioning as measured by neuropsychological tasks, and depressive symptoms [5]. People who had a stroke reported lower quality of life compared to the general population [6, 7] and lower participation in social, vocational and leisure activities [8]. Improvement of emotional well-being during rehabilitation contributed to better health-related quality of life [9]. To foster this, it is important to improve subjective functioning.

Studies that investigate the efficacy of cognitive training typically focus on objective outcome measures. These, however, are not always in agreement with subjective measures [10]. In only about half of the studies a positive relationship between objective and subjective functioning in stroke patients was found [5]. Apparently, objective measures collected in the lab or in assessment rooms do not seem to reflect performance in daily living as perceived by the individual or their surroundings. It is, therefore, important to use both objective and subjective measures.

Impaired self-awareness of cognitive functioning after stroke may, however, make ratings of subjective functioning unreliable. To counter-act this, subjective functioning can additionally be measured by asking a proxy of the stroke survivor. Ratings of subjective functioning have been found to differ significantly between stroke survivors and their proxies [11], although agreement between proxy and stroke survivor may be high for certain subjective measures, such as activities of daily living, and moderate to high for cognitive complaints [5] and quality of life ratings [12].

Efficacy studies that used subjective measures have been inconclusive. Based on a systematic review, computer-based functional retraining of executive functioning was found to result in improvements on several, but not all, subjective measures [13]. To summarize the results of the review, improvements were seen in subjective measures of symptom severity [14], attention [15–17], cognitive functioning [18], participation in social activities [19], and fatigue [15, 20]. No improvements were seen in life satisfaction and self-reported health index [21, 22]. Most studies did not show improvement on measures of depressive symptoms [14, 17, 23, 24], except for one study [25]. Subjective executive functioning improvements were seen after a general cognition training [19], but not after a working memory training [24]. The results were also mixed for subjective quality of life [14, 19] and subjective measures of Instrumental Activities of Daily Living [14, 25]. In the two studies that included proxies, proxies and participants agreed on a positive effect of the training of executive functioning and working memory, but they disagreed on a training effect for attention, as the proxy noted improvement after training that was not reported by the participant [17, 19].

Although these results seem promising, it is not possible to draw firm conclusions that cognitive training improves subjective functioning, because most studies lack proper control groups. Control groups are especially important with respect to subjective measures, because they are more sensitive to placebo effects than objective measures [26]. In the only study that included an active control group, subjective improvements were also seen in this group [19]. Further limitations were that most studies included only a small sample, training duration was generally short, and subjective functioning was commonly assessed with only one outcome measure. Inconsistent findings may also be explained because studies frequently used different outcome measures.

Due to the significance of subjective functioning and quality of life ratings of stroke survivors, we aimed to evaluate the effects of a computer-based cognitive flexibility training on subjective functioning after stroke while accounting for the above-reviewed methodological issues. The training consisted of 58 half-hour sessions focusing on attention, memory, and reasoning. Participants trained at home during 12 weeks. Stroke patients who received this training were compared to an active control group (which received a mock training) and to a waiting list control group. Cognitive flexibility is an executive function that is essential for many everyday life tasks. We, thus, expected that cognitive complaints would be reduced, and that societal participation and activities of daily living would increase to a greater degree after the computer-based training than after the mock training, and that there would be no change in the waiting list group.

## Materials and methods

A detailed description of the design, training tasks, and outcome measures of this study has been published previously [27, 28].

### Participants

**Fig 1:**
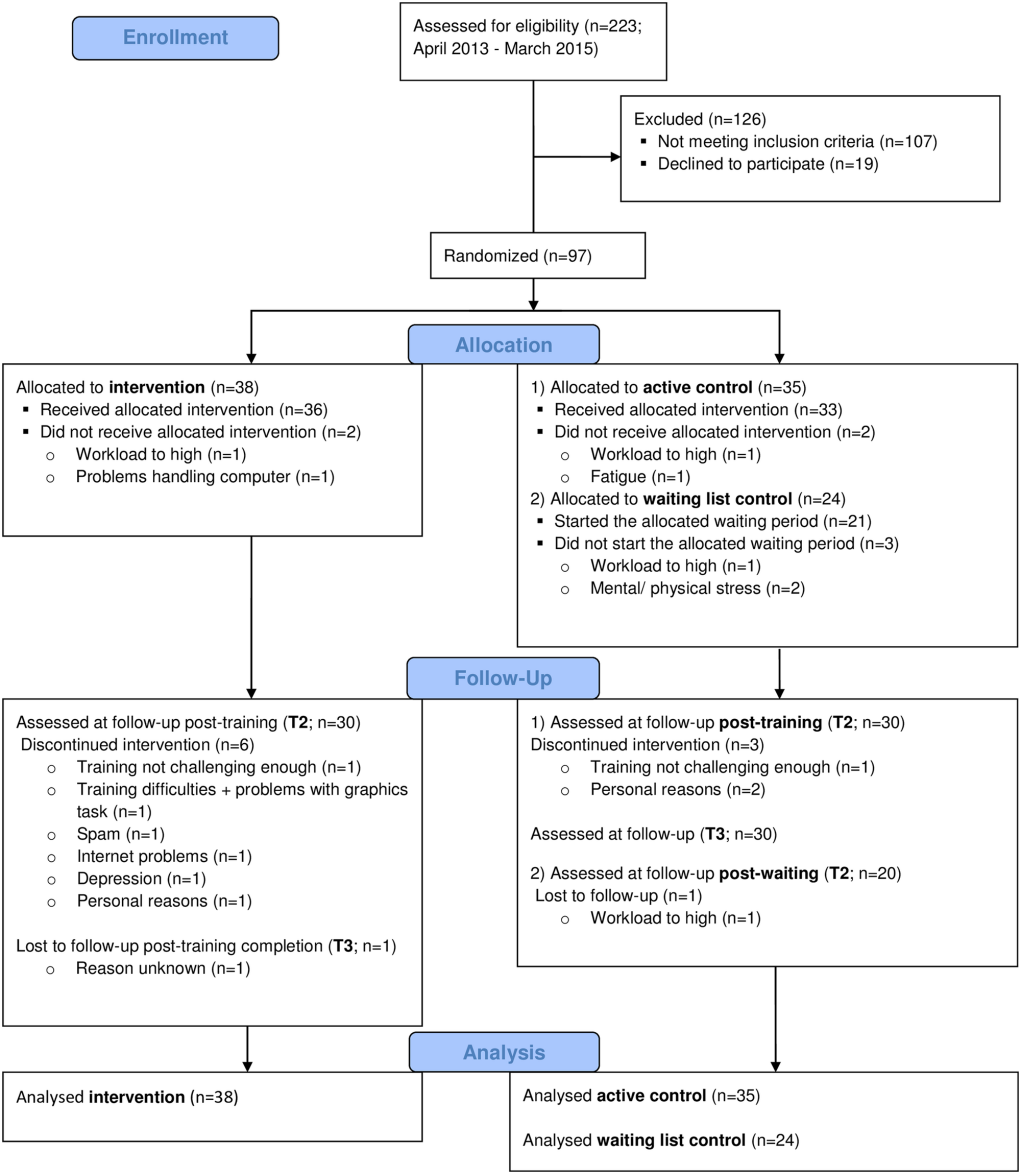
Consolidated Standards of Reporting Trials (CONSORT) flow diagram. T2 = post-training; T3 = 4 weeks after training completion.

Individuals who had a stroke 3 months to 5 years ago and were between 30 and 80 years old were recruited via Dutch rehabilitation centers and patient societies (April 2013—March 2015; last follow-up measurement in November 2015). Participants were selected who had cognitive impairments as testified by medical records, still had cognitive complaints at study entry, and were able to work with a computer. Individuals who had any disease other than stroke that results in severe cognitive impairments, had a history of substance abuse or addiction, or were incapable of executing the training or outcome measure tasks were excluded from the study (see [27] for a full description of the criteria). A schematic overview of the participant flow can be found in Fig 1. Participants included in this study are the same as those in Van de Ven et al (2017) [28].

A-priori sample size calculation suggested a sample size of at least 60 (3x 20) to be able to reveal large effects (*d* = 0.80) in univariate analyses with two groups. Based on an effect size of *d* = 0.56 that was found in a previous training study [29] we strived for a sample of 120 (3 x 40) participants. With the expected attrition rate of 15%, we planned to stop inclusion at 138 participants or when the recruitment period was over.

### Experimental design

The study was a prospective multicenter, double blind, randomized controlled study (RCT). Participants were randomly assigned evenly to one of three groups (intervention, mock training, waiting list) by software [30] that minimized the group differences in age, education, sex, time since stroke, scores on a cognitive screening [31], and level of computer experience. The minimization technique included elements of randomness into the minimization algorithm, to make the prediction to which group a certain participant would be allocated uncertain [32]. Other factors such as rehabilitation site were not considered during the randomization procedure. The groups were coded by the research coordinator. The participants and their proxies were not informed that one of the two training programs was considered to be a mock training, but they were told that we compared the effects of two training programs. The waiting list group was added during the course of the study, but participants before and after this addition did not differ on baseline characteristics.

Several questionnaires were administered online: at baseline (T0), after 6 weeks of training or waiting (T1), after training completion for the training groups or after 12 weeks of waiting for waiting list group (T2), and (for the training groups) 4 weeks after training completion (T3). Participants rated their impression of general recovery on a visual analogue scale (VAS) at T0 and T2. Neuropsychological assessment and MRI scanning were executed at T0 and T2, results of which will be reported elsewhere (see [28] for the results of the objective outcome measures). In compliance with the Helsinki Declaration, the study was approved by the ethical review board of the University of Amsterdam (i.e., Commissie Ethiek voor de Afdeling Psychologie; approved 17^th^ of December 2012) and by the medical ethical review board of the VU University Medical Center, Amsterdam (i.e., the Medisch Ethische Toetsingscommissie Vrije Universiteit Medisch Centrum; approved 9^th^ of July 2013, amendment approved 23^th^ of May 2014). The study was registered before study commencement as Training Project Amsterdam Seniors and Stroke (TAPASS) with the Central Committee on Research Involving Human Subjects Register NL4468502913 (www.toetsingonline.nl). Additionally, to fulfill the World Health Organization Registry criteria it was also registered with the Netherlands National Trial Register NTR5174. The authors confirm that all ongoing and related trials for this intervention are registered.

### Procedures

Participants were asked to select a proxy who was willing to complete questionnaires. The participant came to the University of Amsterdam to receive detailed information about the study, to provide informed consent, to undergo a neuropsychological assessment, and to rate their general recovery on the VAS (T0). The online questionnaires were completed at home by both the participant and the proxy. Depending on the group to which the participant was allocated, they either started training or waited for 12 weeks. The training was performed five times per week and consisted of a total of 58 half-an-hour sessions. Participants of the two training groups were contacted by phone by a neuropsychologist once every week or every two weeks to ask about their training experience and were sent an email as soon as they did not train for two days. The waiting list group was not contacted at all during this period, but received care as usual (which most often meant they did not receive any treatment during the study). After 12 weeks, both the participant and the proxy completed the questionnaires again (T2). A subset of the questionnaires was also completed by the participant after 6 weeks of training or waiting (T1) and in the training groups 4 weeks after training completion (T3). The waiting list group started the intervention training after they completed the questionnaires after the 12 weeks of waiting (T2).

### Intervention materials

Both the intervention and the mock training were carried out by means of a professionally programmed website (www.braingymmer.com) tailored to older adults as well as stroke survivors. Each session lasted 30 minutes in which the tasks of that day were presented in a predefined order and feedback was provided immediately after each task (based on a three-star rating scale) and at the end of each session. The participants were not aware of the training protocol and tasks of the other intervention group. The **cognitive flexibility training** consisted of nine tasks in the domains of attention, memory, and reasoning. A new task of a different domain was presented after 3 minutes. With this fast task switching we aimed to trigger cognitive flexibility. Thus, each session contained 10 tasks. The difficulty of each task was adapted to the participant such that a higher level became available when one out of three stars was achieved; participants had been instructed to go to a higher level whenever two or three out of three tasks were obtained. The **mock training** consisted of four tasks which we considered to train executive functioning only minimally. Participants trained for 10 minutes per task and thus carried out three tasks per session. Task difficulty was not adaptive because participants were instructed to train at a constant level for one to two weeks and could only move to a higher level after this predetermined period. However, participants could (and occasionally did) move to higher levels—thereby disobeying the instructions—as these levels were still accessible.

### Outcome measures

#### Primary outcomes

Subjective cognitive functioning was assessed with the Cognitive Failure Questionnaire [33], which consists of 25 questions completed on a five-point scale with a maximum total score of 100. The CFQ has good psychometric properties including test-retest reliability[34]. It was sensitive to change of a computerized working memory training for stroke survivors [18]. In addition, we created a proxy version that consisted of the same 25 questions asked to the participant.

The Dysexecutive Functioning Questionnaire [35] was used to measure subjective executive functioning. It consists of 20 questions on a five-point scale and the maximum total score is 80. DEX scores could be used to distinguish stroke patients with executive impairments from healthy individuals[36]. In addition, the proxy version of the DEX was used.

Subjective independence level in IADL were measured by the Lawton & Brody IADL scale [37]. The answers on the eight questions on a four- to six-point scale were added up to a total score that ranged from 0 (no problems at all) to maximum 22 (highly impaired). Questions that were answered with ‘not applicable’ or ‘never carried out myself in my life’, were replaced with the average of the remaining items completed by the participant. The IADL scale has good psychometric properties including test-retest reliability [38, 39]. The IADL scale has been sensitive to change after constraint-induced movement therapy in stroke survivors [40]. Furthermore, cognitive impairment before enrollment in stroke rehabilitation could predict IADL scores at 6 months follow-up [41]. A proxy version was created that consisted of the same eight questions asked to the participant.

Subjective quality of life was assessed by the Short Form Health Survey (SF-36) [42]. Total score of mental health was converted to a z-score and corrected for age and sex based on a Dutch norm group [43]. The SF-36 has shown to be valid and reliable in a general population and chronic disease populations in the Netherlands [43].

Rating of participation in society was measured with the Utrecht Scale for Evaluation of Rehabilitation-Participation [44]. It is a 31 items questionnaire that assesses the frequency, restrictions, and satisfaction with participation in social, vocational, and leisure activities. Answers are given on a four- to six-point scale and were averaged and transformed to a 0–100 scale for the three subscales. The answers ‘not applicable’ were not used in the average. As the scores on the three subscales cannot be combined into a total score, we used the restriction subscale as the outcome measure. The USER-P had a good test-retest reliability in a sample that included stroke patients [45].

Scores of all questionnaires were recoded such that higher scores represent better performance. All questionnaires are commonly used in the stroke population for clinical evaluation and science.

#### Secondary outcomes

Subjective recovery after stroke was rated on a 100 mm VAS ranging from 0 (“I did not recover at all”) to 100 (“I recovered completely”).

Subjective cognitive improvement after training or the waiting period was assessed with a four-items exit questionnaire designed for this study. Improvements in overall cognition, attention, memory, and reasoning were rated on a five-point scale ranging from 1 (“I definitely did not improve”) to 5 (“I definitely improved”). Total score was based on the summation of these answers.

Depressive symptoms were assessed with the Hospital Anxiety Depression Scale -depression subscale [46] with a maximum score of 21. The HADS has shown to be a valid measure of emotional distress in people with traumatic brain injury [47].

Subjective level of fatigue was assessed with the Checklist Individual Strength- Fatigue subscale [48] which has a total score between 8 and 56. The test-retest reliability was high for the fatigue subscale in a general population of the Netherlands[49].

Again, scores were recoded such that higher scores represent better performance.

#### Training performance

Performance on the intervention training task was reflected by the levels and scores obtained in the domains attention, memory, and reasoning. The average was taken for the performance on the three training tasks that belonged to the same domain. The maximum total score for each domain was 2000. The mock training tasks did not belong to separate domains and thus the total score was based on the average of the four tasks, and the maximum total score was 900 (see [28] for detailed description of the training performance score).

### Statistical analysis

The main analyses were performed on the data of all participants who started the study (i.e., intention to treat analyses). A repeated-measures MANOVA was performed on the total score on CFQ, total score on DEX, USER-P restriction score, SF-36 mental composite z-score, and total IADL score as dependent variables. Group (intervention, mock training, and waiting list control group) was the independent variable and time-points were before and after the 12 weeks waiting or training period (T0 and T2). In case of a significant time * group interaction effect, post-hoc univariate ANOVAs were performed on the difference score (T2-T0). The analyses were rerun with education, age, and time since stroke as covariates. The IADL scores were not normally distributed thus a Kruskal-Wallis test was used to confirm the outcome of the repeated-measures MANOVA.

Secondary analyses were performed in a similar way with scores on HADS-D, CIS-F, and recovery VAS as dependent variables. The exit questionnaire was only administered after training completion. The total score was not normally distributed; thus the Kruskal-Wallis test was performed to evaluate group differences. The proxy version of the CFQ, DEX, and IADL were analyzed in the same way as the patient version.

In the training groups, Pearson’s correlation coefficients were computed to examine the relation between improvement on the training tasks and change (T2-T0) in the outcome measures. To determine the long-term effects of the training, a repeated-measures MANOVA was performed with CFQ and DEX as dependent variables; group (intervention versus active control) as independent variable; and T0, T2, and T3 as time-points. Post-hoc univariate ANOVAs were performed when the time effect was significant. Analyses were repeated with age, education, and time since stroke as covariates. Exploratory univariate ANOVAs were performed with difference score (T2 –T0) from all outcome measures and group as independent variable.

Missing values were replaced by the method of last observation carried forward (or backward in case the baseline score was missing). In this way, 13.1% of the intention-to-treat data were imputed. Results reported are with outliers because there were no reasons to expect that extreme values were not a reflection of true scores of impaired participants. Analyses were rerun without outliers and whenever results differed they are reported. In these analyses, outliers in the (transformed) raw data were detected by Grubbs’ Extreme Studentized Deviation test [50] and were replaced with the nearest value of another participant in the overall sample. The main analyses were repeated with the participants who completed all follow-up measures and completed at least 50 training sessions (i.e., per protocol analyses).

The scores on the DEX, SF-36, USER-P, HADS, and recovery VAS were transformed because these scores were not normally distributed (see S1 File for formulas). All analyses were performed using SPSS version 22 or higher. P-values < .05 (two-tailed if not mentioned otherwise) were considered significant.

## Results

### Pre-training

After the recruitment period was over, 97 of the 223 potential participants who were screened, met all inclusion criteria and were included in the final analyses (see Fig 1 for participant flowchart including drop-out reasons). At baseline, the three groups did not differ significantly in clinical and demographical variables (Table 1), except for fatigue where the intervention group reported higher levels of fatigue than the active control group (*p* = .02). The proxy reports on cognitive and dysexecutive functioning and independence in IADL did not differ significantly between the three groups.

**Table 1:** Mean (standard deviation) of demographic variables and baseline (T0) outcome measures.

	Intervention group (n = 38)	Active control group (n = 35)	Waiting list group (n = 24)	Sign.
Age (M/median (SD))	57.0/55.0 (9.1)	60.9/ 62.0 (7.5)	61.2/ 60.5 (9.0)	.08
Education (M/median (SD, range))	5.6/6 (1.1, 2–7)	5.6/6 (1.1, 2–7)	5.5/6 (1.3, 2–7)	.95
Sex (% male)	63	66	79	.39^d^
Time since stroke (in months; M/median (SD, range))	28.3/28.0 (16.4, 4.6–59.3)	28.3/29.0 (14.4, 4.1–51.5)	29.1/27.3 (17.0, 5.4–61.1)	.98
TICS (M/median (SD))	34.6/35 (2.1)	34.1/34 (2.8)	34.2/35 (2.4)	.63
Cogn. Rehab. during study (n (%))^b^	2 (5)	5 (14)	2 (12)	.42^d^
Non cogn. rehab. During study (n (%))^b^	13 (34)	14 (40)	4 (24)	.50^d^
*Baseline primary outcomes*				
- Cognitive failure questionnaire^a^	34.2 (13.2)	36.1 (12.4)	36.3 (13.3)	.59
- Dysexecutive Functioning Questionnaire^a^	21.4 (8.6)	23.4 (12.5)	23.7 (9.3)	.73
- Instrumental activities of daily living^a^	3.2 (3.1)	3.6 (3.9)	3.3 (3.6)	.72
- Short Form Health Survey	-0.8 (1.1)	-0.7 (1.1)	-0.5 (0.7)	.76
- USER-P	75.3 (16.6)	71.6 (18.7)	73.4 (16.8)	.91
*Baseline secondary outcomes*				
- Recovery VAS	57.7 (21.9)	58.2 (15.5)	54.4 (26.7)	.91
- CIS-F^a^	39.4 (11.7)	31.5 (12.9)	34.3 (12.3)	.**02**
- HADS-D^a^	6.1 (3.8)	5.3 (3.5)	5.2 (2.4)	.62
*Proxy*^c^				
- Cognitive failure questionnaire^a^	27.6 (14.9)	35.2 (13.3)	30.9 (14.6)	.12
- Dysexecutive Functioning Questionnaire^a^	21.1 (13.8)	27.6 (13.0)	22.8 (16.3)	.18
- Instrumental activities of daily living^a^	3.5 (3.5)	3.6 (4.0)	4.8 (6.3)	.59

*Note*. Bold values are considered significant. Education was based on a 7-point scale (from 1 = unfinished primary school to 7 = university). Sign. = significance; TICS = Telephone Interview for Cognitive Status; Cogn. Rehab. = cognitive rehabilitation; USER-P = Utrecht Scale for Evaluation of Rehabilitation-Participation; VAS = Visual Analog Scale; CIS-F = Checklist Individual Strength- Fatigue subscale; HADS -D = Hospital Anxiety Depression Scale—Depression;

^a^ = lower scores represent better performance;

^b^ *n*_*waiting list*_ = 17;

^c^ = *n*_*intervention group*_ = 30, *n*_*active control group*_ = 31, *n*_*waiting list*_ = 19;

^d^ = p-value based on χ^2^.

### Transfer effect of training

#### Subjective cognitive functioning (primary outcome measures)

The reports by the participants on primary outcome measures revealed a significant time effect (*F*(5,90) = 64.44, *p* = .001, with partial eta squared effect size (*ɳ*_*p*_^*2*^*)* = .20; see Table 2). There was no group * time interaction (*F*(10,182) = 1.35, *p* = .21, *ɳ*^*2*^*ρ* = .07). Post-hoc univariate analyses revealed that the time effect was significant for CFQ (*p* < .001, *ɳ*_*p*_^*2*^ = .18) and DEX (*p* < .01, *ɳ*_*p*_^*2*^ = .08). Thus, all three groups improved in subjective cognitive and executive functioning, including the waiting list group. The time effect disappeared after correcting for age, education level, and time since stroke (*F*(5,87) = 1.30, *p* = .27, *ɳ*_*p*_^*2*^ = .07). However, these variables did not significantly explain any variance, suggesting that the model without these covariates is more valid. In view of the non-normal distribution of IADL scores, we reran the repeated-measures MANOVA without IADL and analyzed IADL with the non-parametric Kruskal-Wallis test, but this did not change the results.

**Table 2:** Mean (standard deviation) and MANOVA of the outcome measures.

measure	Group																		Comparison					
	Intervention group (n = 38)						Active control group (n = 35)						Waiting list group (n = 24)						Time			Time*group		
	Pre-training		Post-training		Δ	*d*	Pre-training		Post-training		Δ	*d*	Pre-waiting		Post-waiting		Δ	*d*	F	p-value	*ɳ*_*p*_^*2*^	F	p-value	*ɳ*_*p*_^*2*^
*Primary*																			F_(5, 90)_	< .**01**	.20	F_(10, 182)_	.21	.07
- CFQ	34.2	(13.2)	31.2	(13.8)	2.9	0.2	36.1	(12.4)	29.3	(11.7)	6.8	0.5	36.3	(13.3)	34.5	(13.6)	1.8	0.1	21.0	< .**001**	.18			
- DEX	21.4	(8.6)	19.6	(9.4)	1.8	0.2	23.4	(12.5)	20.5	(10.7)	2.9	0.2	23.7	(9.3)	22.6	(10.5)	1.0	0.1	8.0	< .**01**	.08			
- IADL	3.2	(3.1)	3.2	(2.8)	0.1	0.0	3.6	(3.9)	3.4	(3.6)	0.2	0.1	3.3	(3.6)	3.7	(3.9)	-0.4	-0.1	0.0	.87	.00			
- SF-36	-0.8	(1.1)	-0.8	(1.1)	0.0	0.0	-0.7	(1.1)	-0.9	(1.2)	-0.2	-0.2	-0.5	(0.7)	-0.6	(0.9)	-0.1	-0.1	0.9	.35	.01			
- USER-P	75.3	(16.6)	74.0	(15.6)	-1.3	-0.1	71.6	(18.7)	76.4	(16.6)	4.7	0.3	73.4	(16.8)	74.3	(17.2)	0.9	0.1	1.3	.26	.01			
*Secondary*																			F_(3, 92)_	.90	.01	F_(6, 186)_	.88	.01
- Recovery VAS	57.7	(21.9)	56.1	(24.0)	-1.6	-0.1	58.2	(15.5)	57.7	(20.5)	-0.4	0.0	54.4	(26.7)	54.8	(27.5)	0.4	0.0						
- CIS-F	39.4	(11.7)	38.4	(13.1)	1.0	0.1	31.5	(12.9)	32.3	(14.3)	-0.8	-0.1	34.3	(12.3)	32.7	(12.9)	1.7	0.1						
- HADS D	6.1	(3.8)	6.1	(3.7)	0.0	0.0	5.3	(3.5)	5.4	(3.8)	-0.1	0.0	5.2	(2.4)	4.9	(2.9)	0.3	0.1						
Cognitive impr.^a^	n.a.		13.6	(3.5)			n.a.		13.3	(3.1)			n.a.		13.1	(3.1)			n.a.			H = .22		
*Proxy*^b^																			F_(3,75)_	.77	.01	F_(6, 152)_	.23	.05
- CFQ	27.6	(14.9)	28.0	(14.7)	-0.4	0.0	35.2	(13.3)	33.5	(13.4)	1.7	0.1	30.9	(14.6)	33.9	(13.9)	-2.9	-0.2						
- DEX	21.1	(13.8)	20.3	(15.2)	0.8	0.1	27.6	(13.0)	26.9	(14.1)	0.6	0.0	22.8	(16.3)	24.6	(15.3)	-1.7	-0.1						
- IADL	3.5	(3.5)	3.1	(3.1)	0.4	0.1	3.6	(4.0)	4.0	(4.0)	-0.4	-0.1	4.8	(6.3)	4.2	(5.7)	0.6	0.1						

*Note*. All scores are total scores where lower scores represent better performance except for SF-36, USER-P, cognitive improvement, and recovery VAS (mm) where higher score reflect better performance; Δ = difference score between pre- and post- measurement recoded in such a way that higher difference score represent improvement; d = Cohen's d (effect size); F was based on Pillai's Trace; H was based on Kruskal-Wallis test; Results were not affected by excluding outliers. Bold values are considered significant and survived Bonferroni-Holm adjustment where appropriate; ɳ_p_^2^ = partial eta squared (effect size); CFQ = Cognitive failure questionnaire; DEX = Dysexecutive Functioning Questionnaire; IADL = Instrumental Activities of Daily Living; SF-36 = Short Form Health Survey- 36; USER-P = Utrecht Scale for Evaluation of Rehabilitation-Participation; VAS = Visual Analog Scale; CIS-F = Checklist Individual Strength- Fatigue subscale; HADS D = Hospital Anxiety Depression Scale—Depression; n.a. = not applicable;

^a^ = analyses based on Kruskal-Wallis test;

^b^ = *n*_*intervention group*_ = 30, *n*_*active control group*_ = 31, *n*_*waiting list*_ = 19.

#### Other subjective functioning (secondary outcome measures)

Results of the secondary outcome measures did not show a significant time effect (*F*(3,92) = 0.45, *p* = .72, *ɳ*_*p*_^*2*^ = .02), or a time * group interaction (*F*(6,186) = 0.40, *p* = .89, *ɳ*_*p*_^*2*^ = .01; see Table 2). Thus, none of the groups improved with respect to depressive symptoms, fatigue, or recovery level. The overall cognitive improvement reported in the exit questionnaire did not differ significantly between groups either (H = 3.07, *p* = .22).

#### Proxy reports

Participants who had a proxy report on at least one of the two time-point were included in the analyses (*n*_intervention_ = 30, *n*_active control_ = 31, *n*_waiting list_ = 19). The above-mentioned significant time effect seen in the participants report was not replicated in the proxy ratings (*F*(3,75) = 0.37, *p* = .77, *ɳ*_*p*_^*2*^ = .02; see Table 2) and the group* time interaction remained non-significant (*F*(6,152) = 1.38, *p* = .23, *ɳ*_*p*_^*2*^ = .05).

#### Relation between improvement on training task and outcome measures

Difference from baseline (T0) to end of training (T2) in the above-mentioned subjective outcome measures was compared to improvement in training tasks in the two training groups. Thirty-six participants who started the intervention training and 33 who started the mock training were included in these comparisons. Even though participants clearly improved on training tasks, correlations between training improvement and change in subjective functioning were weak (*r* ranging from -.28 to .31). Only the correlation of improvement in training tasks with improvement of cognitive functioning (CFQ) in the active control group was significant (*r* = .31, *p* = .04, one-tailed). Overall, the results of proxy reports were similar except for the correlation of improvement in training tasks with cognitive improvement (CFQ), which was weak and not significant (*r* = -.06, *p* = .36).

#### Per-protocol analyses

The main analyses were rerun without participants who dropped out before the T2 measurement (18 participants) or who completed less than 50 training sessions (one intervention and one active control participant). Analyses were based on 77 participants (*n*_intervention_ = 28, *n*_active control_ = 29, *n*_waiting list_ = 20). The 20 participants who dropped out or did not complete the training protocol were not significantly different in baseline subjective functioning from these 77 participants. The only exception was that they were slightly more restricted in their participation (USER-P: *t*(95) = 1.97, *p* = .05) than participants who followed the protocol.

Results from the per-protocol analyses of primary and secondary outcome measures were similar to the intention-to-treat analyses (see S1 Table), thus suggesting that participants who followed the study protocol did not improve more than those who did not.

#### Follow-up

**Fig 2:**
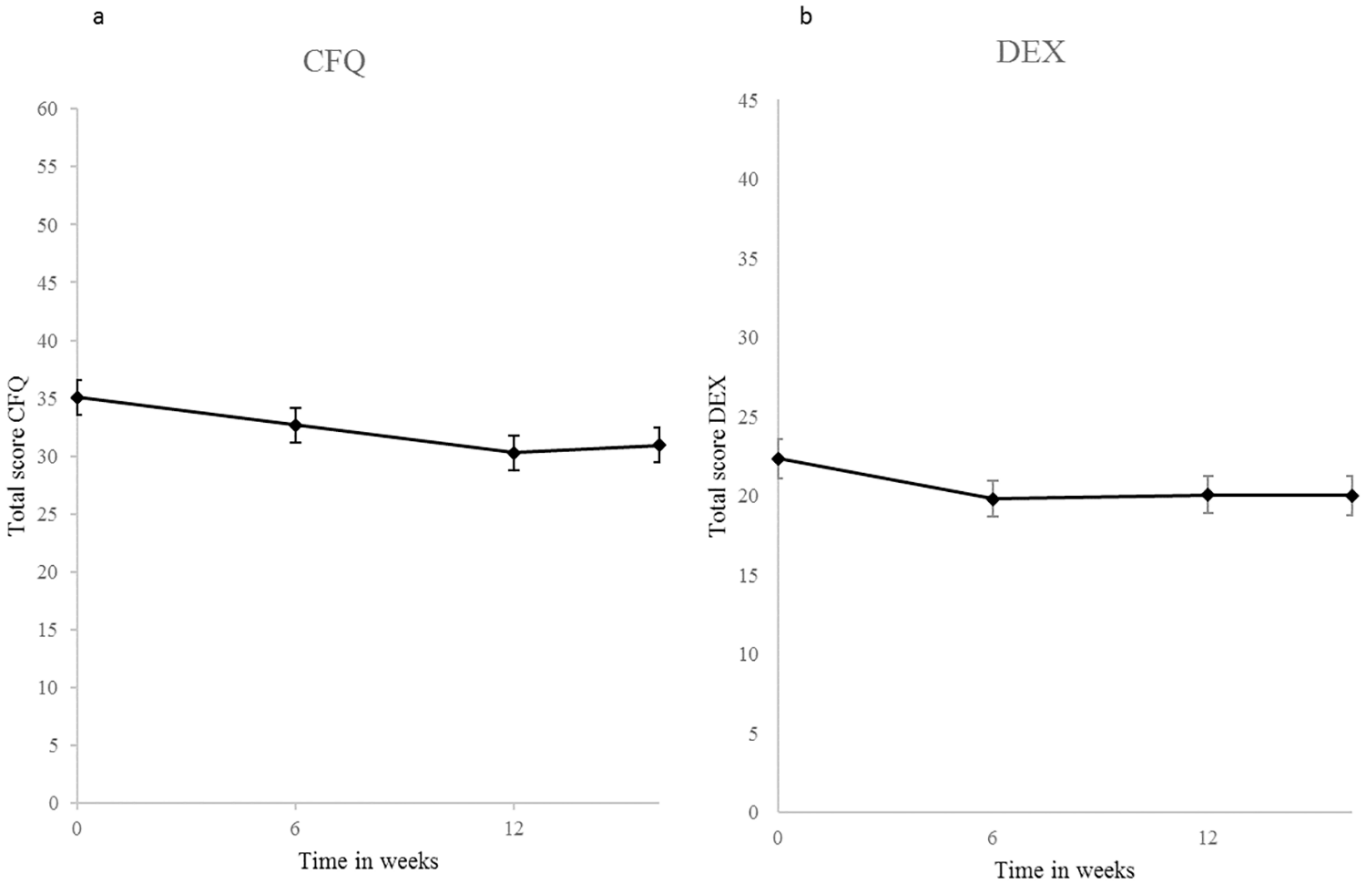
Average subjective cognitive improvement in (a) cognitive functioning and (b) executive functioning of both training groups together. Note. Lower scores represent better performance. Error bars represent standard errors. * = significant improvement (statistical test based on transformed values); CFQ = Cognitive Failure Questionnaire; DEX = Dysexecutive Functioning Questionnaire.

Follow-up measurements were not performed after the waiting period. Consequently, the following analyses refer to the two training groups. Both groups improved significantly over time (F(4, 68) = 5.85, *p* < .001, *ɳ*^*2*^*ρ* = .26) in the CFQ (*p* < .001, *ɳ*^*2*^*ρ* = .21) and the DEX *(p* < .01, *ɳ*^*2*^*ρ* = .09; see Fig 2). Post-hoc pairwise comparisons revealed that follow-up scores were significantly better than baseline scores (T0), but did not significantly differ from the immediate training effect (T2). Thus, scores remained stable after training completion. There was no significant group * time interaction (*F*(4,68) = 1.25, *p* = .30, *ɳ*^*2*^*ρ* = .07). Results from the per-protocol analyses were similar. The significant time effect disappeared after correcting for age, education, and time since stroke. However, none of these covariates explained a significant amount of variance, suggesting that the analysis without covariates is more valid. There was no significant group difference in subjective cognitive improvement (based on the exit list) reported at follow-up (*p* = .06 in intention-to-treat and *p* = .34 in the per-protocol analyses).

#### Exploratory analyses

To confirm that the lack of training effect was not due to insufficient statistical power, we explored the data (post-hoc) with univariate ANOVAs on difference score (T2-T0). The only significant group difference was found for CFQ (*F*(2,94) = 3.31, *p* = .04, *ɳ*^*2*^*ρ* = .07) where the active control group improved significantly more than the intervention (*p* = .05) and waiting list group (*p* = .02). However, this group difference would not remain significant after adjustment for multiple testing. There were no significant group differences when both training groups were pooled and compared to the waiting list control group.

## Discussion

The aim of this study was to examine the effects of computer-based cognitive flexibility training on subjective cognitive functioning after stroke. Results indicate that the computer training did not result in larger improvements than the effect of care as usual. Improvements in all three groups were seen only in subjective cognitive functioning (as measured with the CFQ) and subjective executive functioning (DEX) which remained stable 4 weeks after training completion. These improvements were most likely due to training-unspecific effects such as test-retest effects, placebo effects, or the Hawthorne effect (i.e., the effect of merely participating in a scientific study).

The results are in line with the objective cognitive improvements that were seen in all three groups [28]. Similar to the current report, a time effect was found on three out of five objective executive functioning measures and three out of seven objective cognitive domain scores. This suggests that both the objective and subjective measures were in agreement.

Our results replicate the findings by Spikman et al. (2010), who also found time effects on subjective executive functioning without a superior improvement of the intervention group compared to an active control group. Moreover, Ponsford et al. (1988) found improvements in subjective attention after a waiting period before the start of the training. Thus, training-unspecific factors such as spontaneous recovery and the Hawthorne effect may already result in positive changes.

The time effect on subjective cognitive functioning contrasts with a study using a similar training (though lasting only 8 weeks), which did not result in time or transfer effects [51]. In addition, the absent transfer effect to subjective cognitive functioning differs from the results of a working memory training were subjective cognitive functioning improved more in the experimental group than in passive control group [52]. We cannot rule out, however, that the apparent transfer effect is due to placebo effects, because the study lacked an active control group. Nevertheless, our exploratory univariate ANOVA replicated this latter finding. Namely, we found that subjective cognitive functioning in our active control group improved more than the waiting list group (and intervention group). This may be explained by the fact that our mock training was more adaptive than we had planned, due to participants who managed to achieve higher levels than was allowed based on the training protocol. Therefore, it may have been an effective training after all. However, the group difference in our study was non-significant after correction for multiple testing and should therefore be interpreted with caution.

The lack of improvement in the other questionnaires is in agreement with several studies that also failed to find improvements in IADL [14], depressive symptoms [14, 17, 23, 24], and health related quality of life [21–23, 51]. Spikman et al. (2010) did find improvements in reported quality of life, but this improvement did not differ between the intervention and the active control group. This suggests that effects on general mental health and quality of life are absent or nonspecific, such as placebo effects. Perhaps more frequent and or longer training duration is required to result in far transfer effects in the more general quality of life ratings.

De Luca et al. (2014) did find larger improvements of IADL and depressive symptoms in the intervention group than in the control group, which received care as usual. The training program used was not described in detail, thus it is unclear which elements could have resulted in their positive findings. De Luca et al. included participants who had a severe brain injury 3–6 months earlier, whereas our sample did not report many IADL impairments. Perhaps computer-based training is more effective in improving subjective functioning in a more severely affected population. However, the improvements in IADL found by De Luca et al. might also have been due to spontaneous recovery as improvements were also seen in the control group and their statistical comparison of group differences would not survive adjustment for multiple testing.

Proxies in our study did not report any improvements. This contrasts with the improvements found by Spikman et al. (2010) in both their treatment groups. They included participants with specific executive functioning complaints before the start of study, whereas we included participants with unspecified cognitive complaints. The participants and proxies in Spikman at al. indeed reported worse executive functioning at study entry than the proxies and participants in our study. Thus, more severely affected stroke participants might benefit more from the training. Because Spikman et al. did not include a passive control group, their results may also be due to placebo effects or a regression towards the mean.

There are some limitations to our study that may have affected our results. A general limitation of subjective measures is reactive measurement. Whenever people start to focus on their way of functioning, they may notice more cognitive failures, even though they may have made the same errors before. This would result in increased cognitive dysfunction reports over time, which may have masked any potential training effect.

The relationship between the proxy and the participant (e.g. whether they were partners or siblings) was unknown. It could be that the proxy did not spend enough time in proximity of the participant to notice any changes. The limited reliability of the questionnaires could also have played a role. Sometimes, for unknown reasons, a proxy completed the questionnaire twice at the same time-point, which was possible because the link to the questionnaire remained active, and these scores did not always agree. Because the questionnaires were completed online, it could have taken the proxies more effort to ask for clarifications than when they were completed face-to-face. However, it is unlikely that this led to different answers, as the questionnaires were formulated carefully. Moreover, the test-retest reliability in the proxies of the waiting list group who completed the questionnaires at both time-points (*n* = 16) was acceptable (DEX: *r* = .87, CFQ: *r* = .67, IADL: *r* = .95). Not every participant had a proxy, which may have caused selection bias. Nevertheless, participants with a proxy did not differ on baseline characteristics or time effects from those without a proxy.

Several participants did not complete the follow-up questionnaires (four weeks after training completion, T3). The substituted missing values in the intention-to-treat analyses may have influenced the results. In the intention-to-treat analyses there was a trend-wise significant cognitive improvement (based on the exit list), but this was not confirmed in the per-protocol analyses. The remainder of the follow-up results did not differ between the intention-to-treat and per-protocol analyses, suggesting that the effects on executive functioning and cognitive functioning (based on the CFQ) were valid.

Although not statistically significant, the percentage of participants who received cognitive rehabilitation during the study period differed between groups. This may have biased the training effect. Nevertheless, explorative analyses without participants who received cognitive rehabilitation during the study period did not change the results.

Generally, the effect sizes of change over time were small, which is in agreement with two large RCTs of computer-based training in healthy adults and older adults [53, 54]. One might argue that such small effects are not clinically relevant and not noticeable in daily living.

Possibly, the duration of our training was not sufficient to influence daily living and quality of life. Nevertheless, Westerberg et al. (2007) found improvements on cognitive functioning after 5 weeks of training, whereas Wentink et al. (2016) did not find an effect on the same outcome measure after 8 weeks of training. The relation between training duration and its effect on subjective functioning remains unclear.

The training did not include information on how training improvements could be used in daily living. Patients may benefit from advice on how to apply in daily life what they have learned in these more abstract training tasks. Nevertheless, the training aimed to improve core aspects of executive functioning. When clinically relevant improvements are made, they should generalize to untrained tasks such as daily life activities.

The sample of this study consisted of a heterogenous group of individuals who had a stroke 3 months to 5 years ago. Possibly, the heterogeneity of the sample may have resulted in a large variety of training effects. Previous studies did, however, find improvements in both post-acute stroke samples and chronic stroke samples[18, 19]. Furthermore, time since stroke did not explain a significant amount of variance in any of our analyses. Therefore, we do not assume that this may have affected our results. Future studies are needed to evaluate whether there is an essential time window after stroke in which computer-based retraining is most effective.

One of the strengths of this study is that the sample size was sufficient to detect clinically relevant effects. Lack of statistical power of our main multivariate analyses cannot explain the absence of group differences, because exploratory univariate analyses did not reveal any group difference either, except for CFQ. Second, this study examined everyday life functioning and quality of life. Even though the results are not positive, we hope that future studies continue to include these measures to examine cognitive functioning outside the lab. Another strength is that by including two control groups, we were able to rule out training-unspecific effects such as placebo effects and Hawthorne effect.

Our recommendation for future studies is to measure twice at baseline to avoid reactive measurement and to include objective measures of everyday life functioning. Whenever proxy ratings are included in a study, it is important to register the relationship between proxy and participant and the frequency of contact between the proxy and the participant during the study. Finally, it is important to include proper control groups as subjective measures are prone to placebo effects.

Our study provides insight into whether a computer-based cognitive training, as commercially available, can improve subjective cognitive functioning. Only a few studies have examined the effects of such training on subjective measures of daily living. It is important to include such outcome measures, because subjective functionality in daily living affects wellbeing after stroke. We did find improvements over time in subjective executive functioning and cognitive functioning. Even though these effects were found in all groups, suggesting these are due to training-unspecific effects, this could be beneficial for a better state of mind. Nevertheless, in line with several other training studies, our results did not support the effectiveness of commercial computer-based brain training programs above the effects of care as usual. Our results do not, however, imply that computer-based training programs can never work. More specific and (even) more intense training and prolonged training may have beneficial effects. Future studies may determine whether training programs tailored to the individual are effective. Our general conclusion is that further research is needed before claims about the effectiveness of general brain training can be regarded as evidence-based.

## Supporting information

S1 Table Mean (standard deviation) and MANOVA of the outcome measures of per-protocol analyses. (PDF)

S1 File Subjective brain training effects after stroke. (PDF)

S2 File Protocol approved by medical ethical committee_TAPASS_V1.1. (PDF)

S3 File TAPASS_Subj_CVA_minimal data excl demo info. (SAV)

S4 File CONSORT 2010 checklist_TAPASS_Subjective. (PDF)

## Abbreviations

CFQ: Cognitive Failure Questionnaire
CIS-F: Checklist Individual Strength- Fatigue
DEX: Dysexecutive Functioning Questionnaire
HADS-D: Hospital Anxiety Depression Scale—depression subscale
IADL: Instrumental Activities of Daily Living
SF: Short Form Health Survey
TAPASS: Training Project Amsterdam Seniors and Stroke
TICS: Telephone Interview for Cognitive Status
USER-P: Utrecht Scale for Evaluation of Rehabilitation-Participation
VAS: Visual Analog Scale

## References

[1] van Rijsbergen MWA, Mark RE, de Kort PLM, Sitskoorn MM. Prevalence and profile of poststroke subjective cognitive complaints. Journal of Stroke & Cerebrovascular Diseases. 2015;24: 1823–1831. 25997979 10.1016/j.jstrokecerebrovasdis.2015.04.017

[2] Wilz G. Predictors of subjective impairment after stroke: Influence of depression, gender and severity of stroke. Brain Injury. 2007;21: 39–45. doi: 10.1080/02699050601121996 17364518 10.1080/02699050601121996

[3] Fride Y, Adamit T, Maeir A, Ben Assayag E, Bornstein NM, Korczyn AD, et al What are the correlates of cognition and participation to return to work after first ever mild stroke? Topics in Stroke Rehabilitation. 2015;22: 317–325. doi: 10.1179/1074935714Z.0000000013 26461878 10.1179/1074935714Z.0000000013

[4] Kielbergerova L, Mayer O Jr, Vanek J, Bruthans J, Wohlfahrt P, Cifkova R. Quality of life predictors in chronic stable post-stroke patients and prognostic value of SF-36 score as a mortality surrogate. Transl Stroke Res. 2015;6: 375–383. doi: 10.1007/s12975-015-0418-6 26271301 10.1007/s12975-015-0418-6

[5] van Rijsbergen MWA, Mark RE, de Kort PLM, Sitskoorn MM. Subjective cognitive complaints after stroke: A systematic review. Journal of Stroke & Cerebrovascular Diseases. 2014;23: 408–420. 23800498 10.1016/j.jstrokecerebrovasdis.2013.05.003

[6] Gunaydin R, Karatepe AG, Kaya T, Ulutas O. Determinants of quality of life (QoL) in elderly stroke patients: A short-term follow-up study. Arch Gerontol Geriatr. 2011;53: 19–23. doi: 10.1016/j.archger.2010.06.004 20598382 10.1016/j.archger.2010.06.004

[7] Cerniauskaite M, Quintas R, Koutsogeorgou E, Meucci P, Sattin D, Leonardi M, et al Quality-of-life and disability in patients with stroke. Am J Phys Med Rehabil. 2012;91: S39–47. doi: 10.1097/PHM.0b013e31823d4df7 22193309 10.1097/PHM.0b013e31823d4df7

[8] Blomer AV, van Mierlo ML, Visser-Meily JM, van Heugten C, Post MW. Does the frequency of participation change after stroke and is this change associated with the subjective experience of participation? Arch Phys Med Rehabil. 2015;96: 456–463. doi: 10.1016/j.apmr.2014.09.003 25264108 10.1016/j.apmr.2014.09.003

[9] Katona M, Schmidt R, Schupp W, Graessel E. Predictors of health-related quality of life in stroke patients after neurological inpatient rehabilitation: A prospective study. Health Qual Life Outcomes. 2015;13: 58-015-0258-9. 10.1186/s12955-015-0258-9 PMC4448207 25971254

[10] Maaijwee NAMM, Schaapsmeerders P, Rutten-Jacobs LCA, Arntz RM, Schoonderwaldt HC, van Dijk EJ, et al Subjective cognitive failures after stroke in young adults: prevalent but not related to cognitive impairment. J Neurol. 2014;261: 1300–1308. doi: 10.1007/s00415-014-7346-3 24740819 10.1007/s00415-014-7346-3

[11] Fleming J, Strong J. A longitudinal study of self-awareness: Functional deficits underestimated by persons with brain injury. Occupational Therapy Journal of Research. 1999;19: 3–17.

[12] Oczkowski C, O'Donnell M. Reliability of proxy respondents for patients with stroke: A systematic review. Journal of Stroke & Cerebrovascular Diseases. 2010;19: 410–416. 20554222 10.1016/j.jstrokecerebrovasdis.2009.08.002

[13] van de Ven RM, Murre JMJ, Veltman DJ, Schmand BA. Computer-based cognitive training for executive functions after stroke: A systematic review. Frontiers in Human Neuroscience. 2016;10. 10.3389/fnhum.2016.00150 PMC4837156 27148007

[14] Prokopenko SV, Mozheyko EY, Petrova MM, Koryagina TD, Kaskaeva DS, Chernykh TV, et al Correction of post-stroke cognitive impairments using computer programs. J Neurol Sci. 2013;325: 148–153. doi: 10.1016/j.jns.2012.12.024 23312291 10.1016/j.jns.2012.12.024

[15] Hauke J, Fimm B, Sturm W. Efficacy of alertness training in a case of brainstem encephalitis: Clinical and theoretical implications. Neuropsychological Rehabilitation. 2011;21: 164–182. doi: 10.1080/09602011.2010.541792 21391120 10.1080/09602011.2010.541792

[16] Ponsford JL, Kinsella G. Evaluation of a remedial programme for attentional deficits following closed-head injury. Journal of Clinical & Experimental Neuropsychology: Official Journal of the International Neuropsychological Society. 1988;10: 693–708. 10.1080/01688638808402808 3235646

[17] Ruff RM, Mahaffey R, Engel J, Farrow C, Cox D, Karzmark P. Efficacy study of THINKable in the attention and memory retraining of traumatically head-injured patients. Brain Injury. 1994;8: 3–14. 8124315 10.3109/02699059409150954

[18] Westerberg H, Jacobaeus H, Hirvikoski T, Clevberger P, Ostensson ML, Bartfai A, et al Computerized working memory training after stroke—A pilot study. Brain Injury. 2007;21: 21–29. doi: 10.1080/02699050601148726 17364516 10.1080/02699050601148726

[19] Spikman JM, Boelen DHE, Lamberts KF, Brouwer WH, Fasotti L. Effects of a multifaceted treatment program for executive dysfunction after acquired brain injury on indications of executive functioning in daily life. Journal of the International Neuropsychological Society. 2010;16: 118–129. doi: 10.1017/S1355617709991020 19900348 10.1017/S1355617709991020

[20] Bjorkdahl A, Akerlund E, Svensson S, Esbjornsson E. A randomized study of computerized working memory training and effects on functioning in everyday life for patients with brain injury. Brain Injury. 2013;27: 1658–1665. doi: 10.3109/02699052.2013.830196 24131298 10.3109/02699052.2013.830196

[21] Gray JM, Robertson I, Pentland B, Anderson S. Microcomputer-based attentional retraining after brain damage: A randomised group controlled trial. Neuropsychological Rehabilitation. 1992;2: 97–115.

[22] Lundqvist A, Grundstrom K, Samuelsson K, Ronnberg J. Computerized training of working memory in a group of patients suffering from acquired brain injury. Brain Injury. 2010;24: 1173–1183. doi: 10.3109/02699052.2010.498007 20715888 10.3109/02699052.2010.498007

[23] Gauggel S, Niemann T. Evaluation of a short-term computer-assisted training programme for the remediation of attentional deficits after brain injury: A preliminary study. International Journal of Rehabilitation Research. 1996;19: 229–239. 8910125 10.1097/00004356-199609000-00004

[24] Akerlund E, Esbjornsson E, Sunnerhagen KS, Bjorkdahl A. Can computerized working memory training improve impaired working memory, cognition and psychological health? Brain Injury. 2013;27: 1649–1657. doi: 10.3109/02699052.2013.830195 24087909 10.3109/02699052.2013.830195

[25] De Luca R, Calabro RS, Gervasi G, De Salvo S, Bonanno L, Corallo F, et al Is computer-assisted training effective in improving rehabilitative outcomes after brain injury? A case-control hospital-based study. Disability and Health Journal. 2014;7: 356–360. 24947578 10.1016/j.dhjo.2014.04.003

[26] Schwarz KA, Buechel C. Cognition and the placebo effect—Dissociating subjective perception and actual performance. Plos One. 2015;10: e0130492 doi: 10.1371/journal.pone.0130492 26148009 10.1371/journal.pone.0130492 PMC4493024

[27] van de Ven RM, Schmand BA, Groet E, Veltman DJ, Murre JMJ. The effect of computer-based cognitive flexibility training on recovery of executive function after stroke: Rationale, design and methods of the TAPASS study. BioMed Central NEUROLOGY. 2015;15. 10.1186/s12883-015-0397-y PMC4545547 26286548

[28] van de Ven RM, Buitenweg JIV, Schmand B, Veltman DJ, Aaronson JA, Nijboer TCW, et al Brain training improves recovery after stroke but waiting list improves equally: A multicenter randomized controlled trial of a computer-based cognitive flexibility training. PLOS One. 2017;12. 10.1371/journal.pone.0172993 PMC5336244 28257436

[29] Karbach J, Kray J. How useful is executive control training? Age differences in near and far transfer of task-switching training. Developmental Science. 2009;12: 978–990. doi: 10.1111/j.1467-7687.2009.00846.x 19840052 10.1111/j.1467-7687.2009.00846.x

[30] Saghaei M, Saghaei S. Implementation of an open-source customizable minimization program for allocation of patients to parallel groups in clinical trials. Journal of Biomedical Science and Engineering. 2011;4: 734–739.

[31] Brandt J, Spencer M, Folstein M. The Telephone Interview for Cognitive Status. Neuropsychiatry Neuropsychol Behav Neurol. 1988;1: 111–7.

[32] Saghaei M. An Overview of Randomization and Minimization Programs for Randomized Clinical Trials. Journal of Medical Signals and Sensors. 2011;1: 55–61. 22606659 PMC3317766

[33] Broadbent DE, Cooper PF, FitzGerald P, Parkes KR. The Cognitive Failures Questionnaire (CFQ) and its correlates. British Journal of Clinical Psychology. 1982;21: 1–16. 7126941 10.1111/j.2044-8260.1982.tb01421.x

[34] Bridger RS, Johnsen SAK, Brasher K. Psychometric properties of the Cognitive Failures Questionnaire. Ergonomics. 2013;56: 1515–1524. doi: 10.1080/00140139.2013.821172 23879800 10.1080/00140139.2013.821172

[35] Burgess PW, Alderman N, Wilson BA, Evans JJ, Emslie H. The Dysexecutive Questionnaire In: Wilson BA, Alderman N, Burgess PW, Emslie H, Evans JJ, editors. Behavioural Assessment of the Dysexecutive Syndrome. Bury St. Edmunds, U.K.: Thames Valley Test Company; 1996.

[36] Boelen DHE, Spikman JM, Rietveld ACM, Fasotti L. Executive dysfunction in chronic brain-injured patients: Assessment in outpatient rehabilitation. Neuropsychological Rehabilitation. 2009;19: 625–644. doi: 10.1080/09602010802613853 19199160 10.1080/09602010802613853

[37] Lawton MP, Brody EM. Instrumental Activities of Daily Living (IADL) Scale—Self-Rated Version. Psychopharmacol Bull. 1988;24: 789–791. 3249786

[38] Vittengl J, White C, McGovern R, Morton B. Comparative validity of seven scoring systems for the instrumental activities of daily living scale in rural elders. Aging & Mental Health. 2006;10: 40–47. 16338813 10.1080/13607860500307944

[39] Olazaran J, Mouronte P, Bermejo F. Clinical validity of two scales of instrumental activities in Alzheimer's disease. Neurologia. 2005;20: 395–401. 16217688

[40] Liu KPY, Balderi K, Leung TLF, Yue ASY, Lam NCW, Cheung JTY, et al A randomized controlled trial of self-regulated modified constraint-induced movement therapy in sub-acute stroke patients. Eur J Neurol. 2016;23: 1351–1360. doi: 10.1111/ene.13037 27194393 10.1111/ene.13037

[41] Zinn S, Dudley T, Bosworth H, Hoenig H, Duncan P, Horner R. The effect of poststroke cognitive impairment on rehabilitation process and functional outcome. Arch Phys Med Rehabil. 2004;85: 1084–1090. 15241754 10.1016/j.apmr.2003.10.022

[42] Ware JE, Sherbourne CD. The Mos 36-Item Short-Form Health Survey (Sf-36) .1. Conceptual-framework and item selection. Med Care. 1992;30: 473–483. 1593914

[43] Aaronson NK, Muller M, Cohen PDA, Essink-Bot ML, Fekkes M, Sanderman R, et al Translation, validation, and norming of the Dutch language version of the SF-36 Health Survey in community and chronic disease populations. J Clin Epidemiol. 1998;51: 1055–1068. 9817123 10.1016/s0895-4356(98)00097-3

[44] Post M, van de Port I, Kap B, van Berlekom S. Development and validation of the Utrecht Scale for Evaluation of Clinical Rehabilitation (USER). Clinical Rehabilitation. 2009;23: 909–917. doi: 10.1177/0269215509341524 19717505 10.1177/0269215509341524

[45] van der Zee CH, Priesterbach AR, van der Dussen L, Kap A, Schepers VPM, Visser-Meily JMA, et al Reproducibility of three self-report participation measures: The ICF measure of participation and activities screener, the participation scale, and the Utrecht scale for evaluation of rehabilitation-participation. J Rehabil Med. 2010;42: 752–757. doi: 10.2340/16501977-0589 20809057 10.2340/16501977-0589

[46] Zigmond AS, Snaith RP. The Hospital Anxiety and Depression Scale. Acta Psychiatr Scand. 1983;67. 10.1111/j.1600-0447.1983.tb09716.x 6880820

[47] Whelan-Goodinson R, Ponsford J, Schoenberger M. Validity of the Hospital Anxiety and Depression Scale to assess depression and anxiety following traumatic brain injury as compared with the Structured Clinical Interview for DSM-IV. J Affect Disord. 2009;114: 94–102. doi: 10.1016/j.jad.2008.06.007 18656266 10.1016/j.jad.2008.06.007

[48] Vercoulen JHMM, Bazelmans E, Swanink CMA, Fennis JFM, Galama JMD, Jongen PJH, et al Physical activity in chronic fatigue syndrome: Assessment and its role in fatigue. J Psychiatr Res. 1997;31. 10.1016/s0022-3956(97)00039-3 9447571

[49] Worm-Smeitink M, Gielissen M, Bloot L, van Laarhoven HWM, van Engelen BGM, van Riel P, et al The assessment of fatigue: Psychometric qualities and norms for the Checklist individual strength. J Psychosom Res. 2017;98: 40–46. doi: 10.1016/j.jpsychores.2017.05.007 28554371 10.1016/j.jpsychores.2017.05.007

[50] Grubbs FE. Sample criteria for testing outlying observations. Annals of Mathematical Statistics. 1950;21: 27–58.

[51] Wentink MM, Berger MAM, de Kloet AJ, Meesters J, Band GPH, Wolterbeek R, et al The effects of an 8-week computer-based brain training programme on cognitive functioning, QoL and self-efficacy after stroke. Neuropsychological Rehabilitation. 2016;26: 847–865. doi: 10.1080/09602011.2016.1162175 27184585 10.1080/09602011.2016.1162175

[52] Westerberg H, Klingberg T. Changes in cortical activity after training of working memory—a single-subject analysis. Physiol Behav. 2007;92: 186–192. doi: 10.1016/j.physbeh.2007.05.041 17597168 10.1016/j.physbeh.2007.05.041

[53] Hardy JL, Nelson RA, Thomason ME, Sternberg DA, Katovich K, Farzin F, et al Enhancing cognitive abilities with comprehensive training: A large, online, randomized, active-controlled Trial. Plos One. 2015;10: e0134467 doi: 10.1371/journal.pone.0134467 26333022 10.1371/journal.pone.0134467 PMC4557999

[54] Corbett A, Owen A, Hampshire A, Grahn J, Stenton R, Dajani S, et al The effect of an online cognitive training package in healthy older adults: an online randomized controlled trial. Journal of the American Medical Directors Association. 2015;16: 990–997. doi: 10.1016/j.jamda.2015.06.014 26543007 10.1016/j.jamda.2015.06.014

