# Supplementary material for: The influence of computer-based cognitive flexibility training on subjective cognitive well-being after stroke: A multi-center randomized controlled trial
Source: PLoS One. 2017 Nov 16;12(11):e0187582. doi: 10.1371/journal.pone.0187582 (PMC5690615; doi:10.1371/journal.pone.0187582)
Supplement: S1 Table — (PDF) [file pone.0187582.s001.pdf]

S1 Table. Mean (standard deviation) and MANOVA of the outcome measures of per-protocol analyses.

	Group									Comparison					
	Intervention group (n = 28)			Active control group (n = 29)			Waiting list group (n = 20)			Time			Time*group		
measure	Pre-training	Post-training	Δ	Pre-training	Post-training	Δ	Pre-waiting	Post-waiting	Δ	F	p-value	η_p^2	F	p-value	η_p^2
Primary										$F_{(5, 70)}$	**<.001**	.27	$F_{(10, 142)}$	.17	.09
- CFQ	32.5 (10.8)	28.9 (10.4)	3.7	36.0 (11.2)	27.4 (10.0)	8.6	37.2 (14.3)	34.9 (14.9)	2.3	25.6	**<.001**	.26			
- DEX	21.4 (8.2)	19.6 (9.3)	1.8	23.8 (12.8)	20.2 (10.7)	3.5	24.4 (9.8)	23.2 (11.2)	1.2	7.7	**<.01**	.09			
- IADL	3.2 (3.3)	3.1 (3.0)	0.1	3.3 (3.7)	3.0 (3.3)	0.3	3.3 (4.0)	3.7 (4.2)	-0.4	0.0	.93	.00			
- SF-36	-0.9 (1.1)	-0.8 (1.0)	0.1	-0.6 (1.2)	-0.8 (1.2)	-0.2	-0.6 (0.7)	-0.7 (0.9)	-0.1	0.5	.47	.01			
- USER-P	77.0 (16.1)	74.9 (14.7)	-2.1	74.6 (18.1)	80.0 (14.1)	5.4	73.4 (18.4)	74.4 (18.9)	0.9	0.7	.41	.01			
Secondary										$F_{(3, 72)}$	.77	.02	$F_{(6, 146)}$	.79	.02
- Recovery VAS	60.3 (21.9)	56.8 (24.8)	-3.5	59.5 (14.0)	60.7 (18.1)	1.2	56.9 (25.1)	57.1 (26.5)	0.3						
- CIS-F	38.0 (12.7)	36.5 (14.2)	1.5	30.6 (13.2)	30.9 (14.2)	-0.3	36.2 (11.9)	34.3 (13.0)	1.9						
- HADS D	5.9 (4.0)	6.0 (3.7)	-0.1	5.0 (3.6)	5.1 (4.0)	-0.1	5.1 (2.6)	4.9 (3.1)	0.2						
Cognitive impr.	n.a.	13.4 (4.0)		n.a.	13.3 (3.4)		n.a.	13.1 (3.5)		n.a.			H = 1.0	.60	
Proxy										$F_{(3, 72)}$	.77	.02	$F_{(6, 146)}$	.17	.06
- CFQ	27.9 (14.5)	28.4 (14.2)	-0.5	34.1 (13.0)	32.2 (12.9)	1.9	30.2 (13.8)	33.3 (13.3)	-3.1				2.0		
- DEX	21.4 (13.7)	20.5 (15.2)	0.9	26.5 (11.9)	25.8 (13.2)	0.7	22.5 (15.8)	24.4 (14.9)	-1.8				1.4		
- IADL	3.3 (3.4)	2.8 (2.9)	0.5	3.4 (3.7)	3.8 (3.8)	-0.4	4.7 (6.1)	4.1 (5.5)	0.6				1.9		

Note. All scores are total scores where lower scores represent better performance except for SF-36, USER-P, cognitive improvement, and recovery VAS (mm) where higher score reflect better performance; Δ = difference score between pre- and post- measurement recoded in such a way that higher difference score represent improvement; F was based on Pillai's Trace; H was based on Kruskal-Wallis test; Bold values are considered significant and survived Bonferroni-Holm adjustment where appropriate; η_p^2 = partial eta squared (effect size); CFQ = Cognitive failure questionnaire; DEX = Dysexecutive Functioning Questionnaire; IADL = Instrumental Activities of Daily Living; SF-36 = Short Form Health Survey- 36; USER-P = Utrechtse Schaal voor Evaluatie en Revalidatie - Participatie; VAS = Visual Analog Scale; CIS-F = Checklist Individual Strength- Fatigue subscale; HADS D = Hospital Anxiety Depression Scale - Depression; n.a. = not applicable.
