# Supplementary material for: The influence of computer-based cognitive flexibility training on subjective cognitive well-being after stroke: A multi-center randomized controlled trial
Source: PLoS One. 2017 Nov 16;12(11):e0187582. doi: 10.1371/journal.pone.0187582 (PMC5690615; doi:10.1371/journal.pone.0187582)
Supplement: S1 File — (PDF) [file pone.0187582.s002.pdf]

Supporting Information - Data preparation

Some variables were transformed for data analyses because they were not normally distributed.

The following formulas were used (Tx = timepoint):

DEX=((Max_DEX +1- DEX_Tx)^1.11).

SF36=((4.19 +SF36_Tx)^1.95).

HADSD= 0-Ln(7+HADS_D_Tx).

VAS_Recovery=0-((Max_VAS_Recovery +1- VAS_Recovery_Tx)^0.5).
